# Comparação entre fechamento capsular e capsulectomia em artroplastia total primária de quadril por acesso anterior direto

**DOI:** 10.1055/s-0046-1818620

**Published:** 2026-04-22

**Authors:** Bruno Dutra Roos, Milton Valdomiro Roos, Ezequiel Moreno Ungaretti Lima, Mauricio Domingos Betto, Juan Victor Guzman Gonzales, Henrique Emanuelli Della Méa

**Affiliations:** 1 Serviço de Cirurgia do Quadril, Hospital Ortopédico de Passo Fundo, Passo Fundo, RS, Brasil

**Keywords:** artroplastia de quadril, cápsula articular, estudo comparativo, estudos prospectivos, resultados do tratamento, arthroplasty, replacement, hip, comparative study, joint capsule, prospective studies, treatment outcome

## Abstract

**Objetivo:**

Comparar os resultados clínicos, radiográficos e complicações de pacientes submetidos à cirurgia de reparo ou ressecção capsular em artroplastia total de quadril (ATQ) pela via anterior.

**Métodos:**

Estudo de coorte prospectivo com uma amostra de 232 pacientes submetidos à cirurgia de ATQ em um hospital terciário. Os pacientes foram divididos, conforme a exposição, em um grupo submetido à ATQ com preservação e fechamento capsular e outro grupo submetido à ATQ com ressecção capsular (capsulectomia). Clinicamente, os pacientes foram avaliados de acordo com o Harris Hip Score (HHS), arco de movimento do quadril e presença de sintomas de tendinite do iliopsoas. Radiograficamente avaliou-se a presença de ossificação heterotópica. Avaliou-se também a ocorrência de complicações pós-operatórias.

**Resultados:**

Não se observou diferença estatisticamente significativa em relação ao escore clínico do HHS e arco de movimento do quadril. No grupo fechamento capsular não foram observadas complicações. No grupo capsulectomia, observou-se 7 casos (5,20%) de ossificação heterotópica, com um caso classificado como grau 3 de Brooker, além de encontramos 3 (2,25%) casos de tendinite do iliopsoas e 1 (0,75%) caso de luxação de prótese.

**Conclusão:**

O fechamento capsular na ATQ por via anterior direta demonstrou menor incidência de tendinite do iliopsoas, ossificação heterotópica e complicações como luxação de prótese, em comparação ao grupo capsulectomia.

## Abstract (en)

**Objective:**

To compare the clinical, radiographic, and complication outcomes of patients undergoing capsular repair or resection during total hip arthroplasty (THA) using the anterior approach.

**Methods:**

The present prospective cohort study included 232 patients submitted to THA at a tertiary hospital, who were divided according to exposure. One group of subjects underwent THA with capsular preservation and closure, and the other underwent THA with capsular resection (capsulectomy). Clinical assessment included the Harris Hip Score (HHS), evaluation of hip range of motion, and identification of symptoms indicative of iliopsoas tendinitis. Additionally, a radiological assessment determined the presence of heterotopic ossification, and we evaluated any postoperative complications.

**Results:**

There was no statistically significant difference in HHS and hip range of motion. No patient from the capsular closure group presented complications. In the capsulectomy group, 7 (5.20%) subjects presented with heterotopic ossification, including 1 case classified as Brooker grade 3, besides 3 (2.25%) cases of iliopsoas tendinitis and 1 (0.75%) case of prosthesis dislocation.

**Conclusion:**

Capsular closure in THA using the direct anterior approach resulted in a lower incidence of iliopsoas tendinitis, heterotopic ossification, and complications, such as prosthesis dislocation, compared with the capsulectomy group.

## Introdução

A artroplastia total do quadril (ATQ) é um procedimento cirúrgico amplamente utilizado para melhorar a qualidade de vida de pacientes com doenças degenerativas da articulação coxofemoral. Essa articulação é composta pela congruência entre a cabeça femoral e o acetábulo, estabilizada por estruturas como a cápsula articular, ligamento redondo, lábio acetabular e musculatura adjacente. A cápsula articular contém receptores proprioceptivos e nociceptivos e exerce papel essencial na estabilidade articular, além de atuar como barreira mecânica protetora contra impactos e cargas excessivas. 1 2 Nos últimos anos, o acesso anterior direto (AAD) tem ganhado popularidade devido aos seus benefícios em relação a outras abordagens cirúrgicas, incluindo menor agressão tecidual, recuperação mais rápida e melhor resultado estético. No entanto, sua adoção exige adequada seleção dos pacientes e uma curva de aprendizado por parte do cirurgião. 3 4 5

Alguns autores têm recomendado o reparo da cápsula anterior durante a ATQ por AAD, com base em estudos anatômicos e biomecânicos que demonstram a importância da cápsula na estabilidade da articulação. Ressecções parciais podem aumentar o risco de instabilidade, conforme evidenciado em modelos cadavéricos. 6 7

Diante disso, o objetivo do presente estudo foi o de analisar e comparar os resultados clínicos, arco de movimento do quadril, sintomas de tendinite do iliopsoas, além de resultados radiográficos e complicações de pacientes submetidos ao reparo e à ressecção capsular em ATQ usando o AAD.

## Materiais e Métodos

Trata-se de um estudo de coorte prospectivo. Inicialmente foram selecionados 332 pacientes de hospital terciário, adultos, de ambos os sexos. Os critérios de inclusão foram ter sido submetido à ATQ, por via de AAD e operado por cirurgião único, experiente na via de acesso, membro da equipe de pesquisa, entre o período de 1° de outubro de 2021 a 1° de junho de 2024.

Os critérios de exclusão foram pacientes que realizaram ATQ por fraturas de acetábulo e fêmur proximal, ou revisão de ATQ, presença de infecção vigente, necessidade de reconstruções ósseas, seguimento por menos de 1 ano, ou pacientes que não consentiram em participar da pesquisa.

De acordo com os critérios estabelecidos, 232 pacientes preencheram os requisitos necessários de elegibilidade para a amostra deste estudo. Os participantes do estudo elegíveis foram divididos em dois grupos. O grupo 1 consistiu de pacientes submetidos à ATQ primária por via de AAD com preservação e fechamento capsular, e o grupo 2 de pacientes submetidos à ATQ primária por via de AAD com ressecção da cápsula articular (capsulectomia).

Os participantes do estudo foram avaliados clinicamente de acordo com o Harris Hip Score (HHS) pré-operatoriamente, com 3 meses de pós-operatório, 1 ano de pós operatório e, após o 1° ano, nas consultas anuais de revisão. No período pré-operatório e no último pós-operatório foram aferidos os graus de flexão e rotação externa dos quadris operados, com o uso de goniômetro. O objetivo foi avaliar se havia diferença de flexão e rotação externa do quadril entre os grupos. Na última avaliação pós-operatória, os pacientes também foram avaliados quanto à presença de dor inguinal persistente associada ao exame físico com dor à flexão resistida do quadril, para avaliar a presença de sintoma sugestivos de tendinite do iliopsoas. Na presença destes sintomas, foi realizado exame de ressonância nuclear magnética para confirmação diagnóstica.

Radiograficamente, no último seguimento, os pacientes foram avaliados quanto à presença de ossificação heterotópica de acordo com a classificação de Brooker et al.. 8 Além disso, foram avaliadas complicações com ou sem necessidade de reinternação hospitalar.

Todos os pacientes assinaram de forma individual o Termo de Consentimento Livre e Esclarecido. Fundamentado na Resolução 466/2012 do Conselho Nacional de Saúde e preservará os princípios éticos da beneficência, não-maleficência, justiça e autonomia. O estudo foi aprovado pelo Comitê de Ética e Pesquisa do Sistema CEP/CONEP, sob o n° CAAE: 75628123.7.0000.5342.

O programa utilizado para as análises estatísticas foi o IBM SPSS Statistics for Windows (IBM Corp.), versão 24.0, com extensão PSM ( *propensity score matching* ). Para as estatísticas analíticas de comparação das medidas numéricas entre os momentos pré e pós-operatório, os testes utilizados para comparações entre variáveis categóricas foram o Exato de Fisher e o Qui-Quadrado de *Pearson* . E para comparações entre as variáveis numéricas, por violarem a normalidade (teste de normalidade Kolmogorov-Smirnov), foram utilizados os testes não paramétricos U de Mann-Whitney e Friedman. O nível de significância mínimo adotado foi de 5%.

### Técnica Cirúrgica

O paciente é posicionado em decúbito dorsal em mesa radiotransparente convencional. A incisão normalmente começa aproximadamente 2 cm distal e 2 cm lateral à espinha ilíaca anterior superior e progride distal e lateralmente em direção à borda lateral da patela entre 6 e 10 cm. Após a incisão e hemostasia da pele e do tecido adiposo subcutâneo, o espaço entre o sartório, geralmente de aspecto tendinoso e o músculo tensor da fáscia lata (TFL) é identificado. O primeiro afastador Hohmann tipo cabeça de cobra é posicionado superiormente ao colo femoral, separando o glúteo médio lateralmente. Um afastador tipo Meyerding é usado para retrair a porção reflexa do reto femoral medialmente. Os ramos ascendentes das artérias circunflexas femorais laterais são expostos abaixo da camada fascial profunda do TFL; portanto, é essencial dissecá-lo cuidadosamente e fazer a ligadura ou coagulação desses vasos para diminuir risco de sangramento. Um segundo afastador de Hohmann é colocado medialmente ao colo femoral para retrair o TFL. Após isso, o afastador de Meyerding medial é retirado e um terceiro afastador tipo Hohmann é colocado na coluna anterior do acetábulo para exposição da cápsula articular. Nesse momento, é realizada a ressecção da cápsula articular anterior ou a criação do retalho capsular em forma de “L” ao longo da linha intertrocantérica de base medial, preservando a cápsula anterior. A cirurgia então transcorre de maneira idêntica com colocação dos componentes acetabular e femoral, sendo que nos pacientes submetidos ao fechamento capsular, a cápsula articular é fechada com pontos contínuos de fio poliglicólico 1-0. 9 10 11 12

## Resultados

A idade dos pacientes variou entre 18 e 83, com média de 61,3 ± 12,9 anos, sendo 138 (59,5%) do sexo masculino e 94 (40,5%) do sexo feminino. A maioria teve como diagnóstico coxartrose, correspondendo a 192 (82,8) dos participantes do estudo. O grau de artrose 3 foi o mais frequente, com 191 (82,3) casos, seguido pelo grau 2, com 36 (15,5%), e grau 1, com 5 (2,2%) casos ( Tabela 1 ). O tempo médio de seguimento foi de 19,3 meses (12–34). Do total, 213 (91,8%) das artroplastias eram não-cimentadas, 15 (6,4%) eram híbridas, e 4 (1,7%) eram cimentadas. O grupo 1 (fechamento capsular) foi composto por 99 pacientes (42,7%) com seguimento médio de 14,2 meses (12–18), e o grupo 2 (capsulectomia) por 133 pacientes (57,3%) com seguimento médio de 24,4 meses (14–34).

**Tabela 1:** Caracterização da amostra, conforme grupo de exposição por tipo de artroplastia total de quadril, demonstrada em mediana (IIQ) e número absoluto (%)

Variáveis	Grupo 1	Grupo 2	Valor de *p*
**Idade**	62,0 (55,0–70,0)	63,0 (54,3–70,0)	0,804**
**Sexo**			
Feminino	35 (37,2)	59 (62,8)	0,179 ^¥^
Masculino	64 (46,4)	74 (53,6)	
**Etiologia da osteoartrite**			
Coxartrose	81 (42,2)	111 (57,8)	0,165 ^ɸ^
NACF	5 (45,5)	6 (54,5)	
Sequela de DDQ	2 (22,2)	7 (77,8)	
Protusão acetabular	5 (55,6)	4 (44,4)	
Leg-Perthes	0 (0,0)	3 (100,0)	
Sequela de fratura	6 (75,0)	2 (25,0)	
**Grau de artrose pré-operatório**			
1	2 (40,0)	3 (60,0)	0,236 ^ɸ^
2	20 (55,6)	16 (44,4)	
3	77 (40,3)	114 (59,7)	

**Abreviaturas:** DDQ, doença displásica do quadril; IIQ, intervalo interquartílico; NACF, necrose acetabular da cabeça do fêmur.

**Notas:** *Teste do Qui-Quadrado; **Teste U de Mann-Whitney; ¥ Teste Exato de Fisher; ɸ Teste do Qui-Quadrado de Pearson. **Grupo 1:** Artroplastia total de quadril primária por via de acesso anterior direto com preservação e fechamento capsular. **Grupo 2:** Artroplastia total de quadril primária por via de acesso anterior direto com ressecção da cápsula articular (capsulectomia).

No grupo 1, o HHS médio pré-operatório foi de 52,5 pontos (41,5–62,5), aos 3 meses de 92,5 (80,7–99,9) e no último seguimento de 97,3 (94,6–99,8). Pré-operatoriamente, a média do arco de movimento de flexão do quadril foi de 68,9 graus (50,0–90,0 graus) e de rotação externa de 6,0 graus (0,0–15,0 graus). No último seguimento, o arco médio de flexão do quadril foi de 100,0 graus (90,0–110,0 graus) e de rotação externa de 32,0 graus (10,0 a 45,0 graus). Não houve diagnóstico de casos de tendinite do iliopsoas, presença radiográfica de ossificação heterotópica, ou reinternações por instabilidade protética, infecção ou embolia pulmonar.

No grupo 2, o HHS médio pré-operatório foi de 52,4 pontos (41,0–62,8), aos 3 meses de 92,4 (77,9–99,9), e no último seguimento de 97,0 (94,2–100,0). Pré-operatoriamente, a média do arco de movimento de flexão do quadril foi de 69,7 graus (50,0–90,0 graus) e de rotação externa de 7,2 graus (0,0–20,0 graus). No último seguimento, o arco médio de flexão do quadril foi de 100,3 graus (90,0–110,0 graus) e de rotação externa de 34,0 graus (5,0–45,0 graus). Quatorze participantes tiveram complicações pós-operatórias, todos no grupo ATQ sem fechamento capsular. Portanto, não foi possível aplicar testes estatísticos analíticos devido a presença de zero casos no grupo ATQ com fechamento capsular. Observou-se a presença de três casos (2,25%) de tendinite do iliopsoas. Radiograficamente, houve sete casos (5,20%) de ossificação heterotópica (seis grau 1 e um grau 3 de Brooker). Quanto à necessidade de reinternação, observou-se um caso (0,75%) de luxação protética não-recorrente, um caso de embolia pulmonar (0,75%) e uma infecção superficial (0,75%) com necessidade de desbridamento.

Comparativamente, não se observou diferença estatisticamente significativa entre dois grupos em relação ao HHS e arco de movimento do quadril ( Tabela 2 ). Houve um número maior de casos de tendinite do iliopsoas, ossificação heterotópica e reinternação por complicações no grupo 2.

**Tabela 2:** Harris Hip Score (HHS) e amplitude de movimento nos grupos 1 e 2

Variável	Grupo 1–média (mínimo–máximo)	Grupo 2–média (mínimo–máximo)
**HHS pré-operatório**	52,5 (41,5–62,5)	52,4 (41,0–62,75)
**HHS aos 3 meses**	92,5 (80,7–99,9)	92,4 (77,9–99,9)
**HHS no último seguimento**	97,3 (94,6–99,9)	97,0 (94,2–100,0)
	≤ 0,001*	≤ 0,001*
**Flexão pré-operatória (graus)**	68,9 (50,0–90,0)	69,7 (50,0–90,0)
0,714**		
**Rotação externa pré-operatória (graus)**	6,0 (0,0–15,0)	7,2 (0,0–20,0)
0,097**		
**Flexão no último seguimento (graus)**	100,0 (90,0–110,0)	100,3 (90,0–110,0)
0,448**		
**Rotação externa no último seguimento**	32,0 (10,0–45,0)	34,0 (5,0–45,0)
		0,184**

**Notas:** *Teste de Friedman; **Teste U de Mann-Whitney. **Grupo 1:** Artroplastia total de quadril primária por via de acesso anterior direto com preservação e fechamento capsular. **Grupo 2:** Artroplastia total de quadril primária por via de acesso anterior direto com ressecção da cápsula articular (capsulectomia).

## Discussão

Nesta coorte prospectiva, as principais complicações cirúrgicas foram ossificação heterotópica e tendinite do iliopsoas. Os resultados demonstram a importância que a cápsula articular tem como protetor anatômico entre o componente protético acetabular e o iliopsoas.

Embora não tenha sido observada diferença significativa na taxa de luxação entre os grupos no atual estudo, Miranda et al. 13 realizaram uma revisão sistemática com 31 artigos, demonstrando que os pacientes submetidos ao reparo capsular apresentaram menor taxa de luxação (0,65%) quando comparados com os pacientes submetidos à capsulectomia (3,06%). Em outro estudo, Kanda et al. 14 não evidenciaram casos de luxação de prótese em pacientes com preservação da cápsula articular em um acompanhamento de 6 meses. Imagama et al. 15 demonstraram que a sutura capsular anterior reduz força de tração axial produzida pelo cirurgião no intraoperatório.

Por outro lado, Vandeputte et al., 16 em um estudo prospectivo randomizado de coorte com 219 quadris, em que foram comparadas as técnicas de preservação e ressecção capsular, concluiram que não houve diferença estatisticamente significativa entre as variáveis analisadas.

McLawhorn et al. 17 publicaram um estudo prospectivo com 32 pacientes, dos quais 17 foram submetidos ao reparo capsular e 15 à capsulectomia. Após 1 ano, os quadris foram avaliados com exame de ressonância magnética, e observou-se que os pacientes submetidos ao reparo capsular apresentavam cápsula anterior intacta, enquanto os pacientes submetidos à capsulectomia evidenciavam defeito persistente em 27% dos casos.

## Conclusão

A realização da preservação e fechamento capsular na ATQ por via anterior direta demonstrou menor incidência de complicações clínicas e radiográficas, particularmente em relação à tendinite do iliopsoas e à ossificação heterotópica, quando comparado ao grupo capsulectomia. A função articular, avaliada pelo HHS, apresentou resultado semelhante em ambos os grupos.
